# Obtaining new brewing yeasts using regional Chilean wine yeasts through an adaptive evolution program

**DOI:** 10.3389/fmicb.2025.1599904

**Published:** 2025-06-16

**Authors:** Ángela Contreras, Manuel Villalobos-Cid, Cristian Valdés, Carlos A. Villarroel, Felipe Castro, Ignacio Farías, Gustavo Lorca

**Affiliations:** ^1^Center for Biotechnology of Natural Resources, Faculty of Agricultural and Forestry Sciences, School of Biotechnology, Universidad Católica del Maule, Talca, Chile; ^2^Departamento de Ingeniería Informática, Facultad de Ingeniería, Laboratory of Artificial Intelligence applied to Bioinformatics, Universidad de Santiago de Chile, Santiago, Chile; ^3^Centro de Investigación de Estudios Avanzados del Maule (CIEAM), Vicerrectoría de Investigaciones y Posgrados, Universidad Católica del Maule, Talca, Chile; ^4^Departamento de Biotecnología, Kayta, Santiago, Chile; ^5^Faculty of Agricultural and Forestry Sciences, School of Agronomy, Universidad Católica del Maule, Talca, Chile

**Keywords:** adaptive evolution, Brewer’s yeasts, regional Chilean wine yeasts, beer production, genetic improvement

## Abstract

Beer consumption has increased worldwide, positioning it as the most consumed alcoholic beverage on the market. *Saccharomyces cerevisiae* brewing yeasts have specific genetic characteristics that allow them to survive in malt wort using maltose and maltotriose as the principal carbon source. However, metabolizing these sugars is challenging for non-brewery *Saccharomyces* strains under typical brewing conditions, which involve high osmotic stress and low temperatures. These conditions restrict beer producers to a limited range of yeast strains, increasing their cost and contributing to beer flavors uniformity. Here, we performed an adaptive evolution process to improve the fermentative capacities of *S. cerevisiae* winemaking yeasts isolated from Chilean vineyards to allow their use in brewing. Initially, we screened 50 strains of viticultural origin collected from different areas of Chile. Five strains were selected based on their fermentative capacities, sugar consumption, flavor and aroma, after which were subjected to an adaptive evolution process of 600 generations. We obtained an evolved strain that was able to consume maltose and maltotriose, growing in very high gravity wort (29°P) and at low temperatures (18°C) without shaking. We used DNA sequencing to examine genome changes that could explain the superior beermaking phenotype of the evolved strain. We found that the evolved strain completely lost a parental genome and showed aneuploidies, resulting in gene copy number variations. Interestingly, duplications in genes related to maltose metabolism (*IMA1*, *MAL13* and *MAL11*) were observed. Moreover, we also found 160 genes that lost a copy in the evolved strain, of which three showed complete loss: *FLO5*, *PAU*8, and *SEO1*. These genes are related to wine yeast survival under the stress conditions of grape must (lower pH, higher glucose and ethanol concentration than wort). Our results show a successful application of high stress levels to evolve regional winemaking strains to improve their fermentative traits for the brewing process. Applying this method will make it possible to obtain yeasts that can carry out alcoholic fermentation in wort without having to buy unique strains called “brewing yeasts.”

## 1 Introduction

Beer is the most consumed alcoholic beverage in the world (Prasannan, 2018). In 2018, 1.94 billion hectoliters of beer were produced worldwide (Conway, 2019, 2020). The global beer market’s is projected to reach 710.89 USD billion by 2025, and this forecast also indicates an increase in customer preference for high-quality beers (Marshall, 2020). Due to the high demand for beer, brewers have been introducing new processes such as the use of very high gravity wort (>25°P) to shorten production times to make dilutions and assembles (Abbott et al., 2020; Puligundla et al., 2020; Russell and Stewart, 1998; Stewart, 2010; Liti et al., 2009). Also, brewers have started to produce beers with innovative flavor, color and foam, which allows them to differentiate their product from other beers (Behr et al., 2020; Stewart, 2016).

A key factor to improve the brewing process is yeast, which consume sugar present in wort to carry out the alcoholic fermentation, producing ethanol, CO_2_, and modulating beer flavors and aroma (Day et al., 2002; Hough et al., 1982). Brewing yeast mainly belongs to the *Saccharomyces cerevisiae* species and *Saccharomyces pastorianus* interspecies, which produce lager and ale beer, respectively (Behr et al., 2020). *S. cerevisiae* strains ferment wort at elevated temperatures to produce top-fermented beer styles, such as ales beer. In contrast, *S. pastorianus* yeasts are used to produce bottom-fermented beer styles, such as lager, stout, and others. Lager yeasts have the capacity to flocculate, and therefore, can precipitate to the bottom of the fermenter, facilitating their elimination at the end of fermentation. Additionally, they can ferment in a wide range of temperatures, including low temperatures (Verstrepen et al., 2003; Behr et al., 2020). *S. cerevisiae* is a pure species whereas *S. pastorianus* is a hybrid of *S. cerevisiae* and *Saccharomyces eubayanus* (Hutzler et al., 2023). In general, most brewers use commercial yeast strains belonging to these two types of yeasts. They are usually isolated from beer production places, and can consume maltose and maltotriose efficiently under brewing conditions (Hornsey, 1999).

However, there are few options for beer differentiation because the current commercial strains of *Saccharomyces*, and its progenies, are insufficient to provide new characteristics to beer. For this reason, it is necessary to diversify the supply of new and improved strains that can withstand different stresses commonly found in industrial beer production (Aquilani et al., 2015; Alperstein et al., 2020; Gibson et al., 2020; Molinet and Cubillos, 2020).

Generally, the usual stress conditions in beer production are mainly low temperature, low oxygenation, and a complex carbon source that is difficult to metabolize by non-brewing yeast. The fermentation process is performed at temperatures ranging from 5°C to 20°C, whereas *S. cerevisiae* optimum growth is within 25–30°C. Moreover, beer production is carried out without shaking, limiting oxygen availability. The main carbon sources available in wort are maltose (60–70%) and maltotriose (15–20%), where maltotriose is more difficult to be consumed for yeasts compared to maltose (Day et al., 2002; Hough et al., 1982).

Nowadays, several yeast bioprospecting studies have been carried out, isolating wild strains from non-conventional environments to select strains with high genetic variability for the beer industry (Duan et al., 2018; Peter et al., 2018; Kang et al., 2019; Pontes et al., 2020; Molinet and Cubillos, 2020). Besides, several studies have been focusing on: (1) the use of non-conventional yeast species such as *Dekkera/Brettanomyces*, *Wickerhamomyces anomalus*, and *Torulaspora delbrueckii* in beer production (King and Dickinson, 2000); and (2) the use of *S. cerevisiae* strains isolated from non-brewing areas (Rossi et al., 2018). However, traditional commercial yeasts are still more efficient than these alternative yeasts, because they consume the sugars present in wort faster, including at low temperatures (Liu and Hui Quek, 2016; Rossi et al., 2018; Toh et al., 2020). A common problem in brewing is associated with the incomplete use of maltotriose, affecting beer quality due to: (1) high residual sugar, (2) lower flavor complexity, and (3) microbiological instability (Duval et al., 2010; Nascimento and Fonseca, 2019; Stambuk et al., 2006). Several genetic engineering studies have been focused on improving yeast brewing performance (Blieck et al., 2006; Bond et al., 2004; Brejning et al., 2005; Gibson et al., 2008; Higgins et al., 2003; James et al., 2003; Joubert et al., 2001; Kobi et al., 2004; Minato et al., 2009; Mizuno et al., 2006; Nakao et al., 2009; Olesen et al., 2002; Pope et al., 2007; Saerens et al., 2010; Yoshida et al., 2008). Nevertheless, consumers have not accepted the genetically modified strains obtained from genetic engineering, and different countries have limited their distribution through several regulations (Bitew and Andualem, 2024).

Likewise, adaptive evolution has become a non-GMO alternative to improve organoleptic differentiation (Çakar et al., 2012). It can be defined as the process that generates gradual and permanent changes in an organism’s genome, increasing its adaptation, survival rate and reproducing abilities under different environments (Kull, 2013). Specifically, the adaptive evolution process for yeast involves the use of one or more stressors during yeast culture (Gibson et al., 2020). These stressors generate selective pressure that can be constant or gradually increased, allowing the fittest strains to survive. An adaptive evolution program requires parental strains with high genetic variability to obtain different phenotypes in their offspring (Reed and Frankham, 2003). *S. cerevisae* has a wide presence worldwide living in diverse niches. Therefore, the strains of this species show high genetic and phenotypic variability (Boynton and Greig, 2014; Gibson and Liti, 2015). Despite this high variability, brewing yeast are usually genetically similar, probably because they have adapted to similar stressful environments. During beer production, brewing yeasts are continuously recycled after each fermentation increasing domestication while reducing genetic variability (Steensels and Verstrepen, 2014). Instead, winemaking yeast strains are typically used only once, after which they are often released into the surrounding agricultural area. This practice might increase yeast genetic diversity due to their interaction with other microorganisms (Molinet and Cubillos, 2020). Therefore, wine yeasts that have locally adapted to the winemaking region (hereafter referred to as “regional” yeasts) may retain higher genetic variability, potentially enhancing their ability to adapt to new stressful environments (Marsit and Dequin, 2015; Martínez et al., 2007; Mortimer et al., 1994; Sipiczki, 2010). Hence, here we used yeasts isolated from Chilean vineyards to contribute to the genetic variability for an adaptive evolution program conducted in high gravity beer wort. After 600 generations, we obtained an evolved strain that was able to ferment very high gravity wort (29°P), exhibiting maltose and maltotriose consumption, at a low temperature and without agitation. Beers produced using this strain showed high attenuation and good sensory properties. Furthermore, the evolved and parental strains were subjected to genome sequencing, which showed that the evolved strain lost a complete genome copy from the heterozygous diploid parental. However, flow cytometry indicated that the evolved strain remained as a diploid, suggesting autodiploidization after sporulation.

## 2 Materials and methods

### 2.1 Microorganisms and media

We used fifty regional *S. cerevisiae* isolates from 12 different viticulture places in Chile, which are part of the Microorganism Culture Collection of the Kayta company in collaboration with LAMAP-University of Santiago of Chile (Supplementary Table S1; Supplementary material A1). In addition, strains isolated from Argentine Patagonia (part of the LAMAP-culture collection) were included as a reference, due to their brewing capacities. Cryogenically preserved (−80°C) strains were cultured and maintained on YPM medium (3 g/L malt extract, 3 g/L yeast extract, 5 g/L peptone, 10 g/L glucose, 16 g/L agar) plates and stored at 4°C.

### 2.2 Isolation and verification of autonomous character and species of the yeast strains used

Samples were taken at the end of the fermentations carried out with grapes from these different viticulture places in Chile. They were diluted in 1 moL/L sorbitol and plated on YM agar (yeast extract 3 g/ l, malt extract 3 g/L, peptone 5 g/L and glucose 10 g/L) supplemented with ampicillin (100 mg/L). The taxonomic identity of these yeast isolates was pre-identified by growth on lysine and WL medium (Oxoid, Basingstoke, United Kingdom). Furthermore, yeast identification at the genus, and/or species, level was carried out by a 5.8S-ITS RFLP analysis as described Esteve-Zarzoso et al. (1999). To determine the autochthonous character of the selected yeasts, we used the Random Amplified Polimorphic DNA (RAPD) molecular technique, considering eight random primers (Echeverrigaray et al., 2000). We analyzed gel electrophoresis imaging using the Gel Analyser™ program, which allowed us to identify electrophoretic bands in each strain and constructing a dendrogram (Supplementary Figure S1; Supplementary material A1). At this stage, we analyzed L261, L170, 8jj, L169 and L249, and the commercial strains Fermicru XL™ (wine), Lalvin™ EC1118 (wine), Lallemand Nottingham™ (beer), WLP001™ (beer) and WLP004™ (beer). The figure shows that the *Saccharomyces* strains analyzed had a higher similarity (short distance) with the Fermicru XL strain. This strain has been confirmed as an indigenous Chilean wine strain. On the other hand, a higher distance with the commercial strains was observed (Supplementary Figure S1; Supplementary material A1).

### 2.3 Screening for the yeast of the genus *Saccharomyces* in YPMM broth

Each strain was cultured in a medium where the primary carbon sources were maltose and maltotriose. We generated a pre-inoculum of each strain, cultivated in YPM broth, which was inoculated at 1 × 10^6^ cells /ml in 50 mL of YPMM broth (0.5% peptone, 0.5% yeast extract, 2% maltose and 1% maltotriose), using 100 mL Erlenmeyer flasks. The cultures were incubated at 28°C for 24 h with shaking (120 rpm). Maltose and maltotriose consumption was evaluated as a selection phenotype; the determination was carried out by HPLC, as described below.

### 2.4 Fermentative behaviour analysis of the selected strains in synthetic wort

At this stage, synthetic wort was prepared using malt extract with a concentration of 150 g/L, generating 1,060 g/mL of final density at pH 5.4. Wort was sterilized at 121°C for 15 min. We prepared the cultures in 250 mL Erlenmeyer flasks with gas outlets. We inoculated the culture volume of 100 mL considering 4 ×10^6^ cells/ml, this cell concentration allows the culture to develop. Cell viability was verified with methylene blue. Before inoculation, we aerated the wort by shaking for 5 min. All cultures were fermented for 7 days at 18°C without stirring.

### 2.5 Adaptive evolution applied to the selected strains

**Figure 1:**
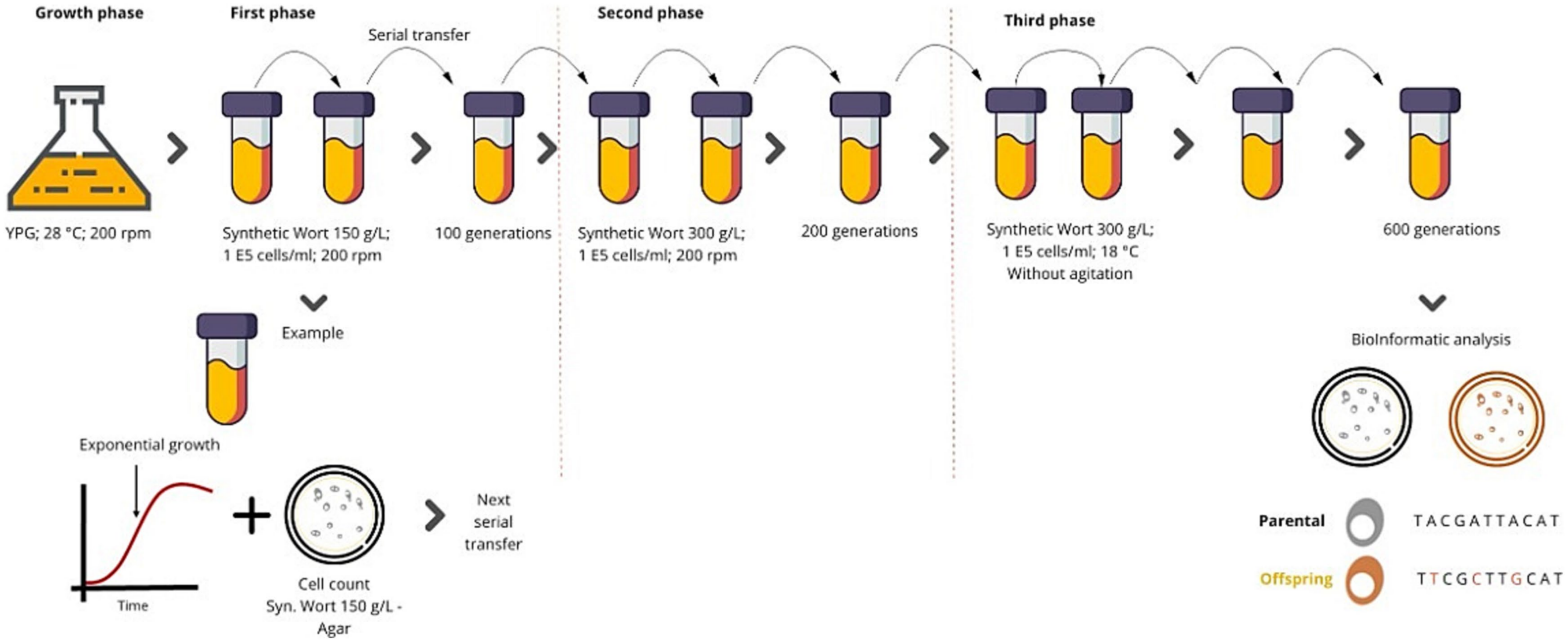
Adaptive evolution protocols. The adaptive evolution protocol consisted of three phases. 1st) The selected strains were grown using wort with 150 g/L of malt extract, they were incubated at 20 ° C and 200 rpm of agitation, and every time the cultures reached half of the exponential phase, they were passaged to fresh medium. 2nd) A must containing 300 g/L of malt extract was used, and the cultures were incubated at 20 ° C and 200 rpm of agitation, repeating the passages. 3rd) A wort of 300 g/L was used and the cultures were incubated at 18 ° C without agitation. The concentration of cells in each transfer was 1*10^5^ cells/ml. Samples were taken during each passage, and it were inoculated in Petri dishes with malt extract agar at the corresponding concentration.

The process was started by inoculating each yeast strain into an YPG (3 g/L yeast extract, 5 g/L peptone, and 10 g/L galactose) culture broth. It was necessary to use this medium to activate the metabolic pathways related to the consumption of maltose and maltotriose early (Higgins et al., 2001). In this YPG broth, each yeast was cultured for 48 h with shaken (200 rpm) at 28 ° C. We performed an adaptive evolution process, which comprised three phases. For the first phase, we used synthetic wort with a concentration of malt extract of 150 g/L (maltose and maltotriose concentrations around 50 g/L and 30 g/L, respectively). This wort had a final density of 1.060 g/mL. At the beginning of the process, the volume of inoculum was 1 × 10^5^ cells /ml, which was grown at 20°C with 200 rpm of agitation. During the evolution process, we monitored all cultures to collect cells in the middle of the exponential growth phase to inoculate 1 × 10^5^ cells/ml into fresh wort of equal concentration again. This activity was sequentially replicated many times to reach 100 generations (depending on the doubling time observed when monitoring each growth). For the second phase, we carried out the same protocol using wort with a malt extract concentration of 300 g/L (maltose and maltotriose concentrations around 120 g/L and 40 g/L, respectively). This wort had a final density of 1.125 g/mL (~29°P). This phase was carried out over 100 more generations (Figure 1). The cells were transferred in each passage, isolating colonies at each stage. Subsequently, a third phase of evolution was performed, where the culture temperature was reduced to 18°C and agitation was limited. We repeated this activity until a total of 600 generations were reached (Figure 1). Culture samples were taken for each repetition, and a cell count was performed in a Neubauer chamber. In addition, to monitor the cell viability, a pour-plating was carried out on soft agar plates, using wort at the same concentration of the liquid wort used. At the end of each evolution phase, between 3 and 10 colonies were taken per plate and were evaluated in YPM (yeast extract, peptone and maltose), using a maltose concentration of 120 g/L. For this, multi-well plates were used, where growth was evaluated through optical density quantification. These quantifications were carried out using Tecan^®^ equipment (Männedorf, Switzerland). The microplates were inoculated using six replicates at a concentration of 1 × 10^5^ cells /ml; they were incubated at 20 ° C with or without shaking. In the adaptive evolution process, the initial yeast strains were called “parental” and the strains obtained at the end of the evolution process were called “offspring” or “evolved strain.”

### 2.6 Determination of fermentation kinetics of each evolved yeast using synthetic wort supplemented with high maltose concentration

As detailed earlier, the evolution program consisted of isolated colonies using synthetic wort agar plates at each stage. Then, different maltose concentrations were used (150 or 300 g/L) depending on the stage. The colonies obtained for each strain during this process were evaluated for fermentation kinetics phenotypes in YPM broth (yeast extract, peptone, and maltose), using 120 g/L maltose concentration. This evaluation was carried out in 96-well Cell Culture Plates (Thermofisher, United States), using a microplate reader (Tecan, Swiss).

### 2.7 Fermentation of natural wort by *Saccharomyces cerevisiae*

The selected “evolved” strains obtained from the adaptive evolution process were evaluated using Pale Ale wort. The strains were inoculated in wort at a cell concentration of 5×10^6^ cells / ml in triplicate, using a 10-liter reactor (Biocl, Chile). The cultures were incubated at 18°C without agitation for 7 days. Then, they were decanted at 4°C for 5 days, and then 300 mL were packaged in 330 mL amber beer bottles. Cells of evolved strains used for inoculation were obtained directly from their propagation in YPM-120 broth (3 g/L yeast extract, 5 g/L peptone, 10 g/L glucose, 120 g/L malt extract). They were incubated at 18°C for 2 days. On the other hand, cells of parental strains used for inoculation were obtained from an adaptation process in which maltose concentration was increased from 30 to 120 g/L. After this process, incubation temperature was decreased from 25°C to 18°C.

### 2.8 Pale Ale wort elaboration

We used Pale Ale wort and Kent Golding hops because their combination makes a neutral beer, which allows us to evaluate the contributions of flavor and aromas provided by the yeast. We mixed Pale Ale malt grain with water (1:4) previously heated to 70°C. Next, the mixture was heated at 65°C for 90 min with stirring every 15 min. After 30 min we included Kent Golding hops (0.8 g/L) was added to the mixture. Then, we filtered the mixture, separated the broth from the malt grain, and heated this new mixture at 100°C for 30 min. Finally, the wort was rapidly cooled, filtered and stored at 4°C. The density and pH of the wort were 1,050 g / ml and 5.1, respectively.

### 2.9 Analytical techniques

Yeast growth was followed spectrophotometrically by measuring the absorbance at 600 nm. Viable cell counts were determined by plating on WL agar, and plates were incubated at 28°C for 2 days. Moreover, the viability of each inoculum was confirmed by cell counting in a Neubauer chamber, using methylene blue. Flocculation was determined by Helm’s test (Bendiak, 1994). Ethanol, glucose, fructose, glycerol, maltose, and maltotriose were quantified by high-performance liquid chromatography (HPLC), using a Shodex sugar SH1011 column (Phenomenex, United States). A Shimadzu Prominence HPLC equipment (Shimadzu, United States) was used. In addition, 5 mM H_2_SO_4_ was used as the mobile phase, in an isocratic flow of 0.6 mL / min, with the oven temperature set at 5°C.

### 2.10 Statistical analysis

Differences between measurements were determined using the Mood test (Gibbons and Chakraborti, 2011) using the statistical software Statgraphics Centurion (v5.0). We observed significant differences when *p*-values were less than 0.05.

### 2.11 Sensory analysis

Two sensory analyses were carried out during this study. The first analysis was performed during “fermentative behaviour analysis of the selected strains in synthetic wort,” where the wort fermented was evaluated by five judges with Beer Judge Certification Program (BJCP) certification. The evaluation considered aroma and taste as quality parameters. A score scale was used to qualify, a score of 1 indicated “I dislike” and score 5 indicated “I like a lot.”

The second analysis was performed at the end of the study, in which beer was produced on a pilot scale. We prepared 10-liter reactors (Biocl, Chile) with Pale Ale wort for each inoculated yeast in triplicates. The yeasts used were (1) Lallemand Nottingham™ (brewer’s yeast) and (2) L261col5max (yeast evolved). The L261 (parent) strain was not evaluated because it did not grow in wort. Additionally, fermentation blanks, corresponding to Pale Ale wort, were included and prepared under the same conditions as the samples but not inoculated with yeast. Therefore, 300 mL were packaged in 330 mL amber beer bottles to be later evaluated by 25 judges with and without training.

A hedonic test was performed with a score range of 1 to 5. The details of this scale are shown in Supplementary Table S2 and Supplementary material A1. Attributes evaluated were appearance, color intensity, beer clarity, foam consistency, aromas, esters, flavor complexity and beer body.

In addition, a preference test (Ranking) was carried out, where we asked the judges which beer they liked the most.

### 2.12 Beer volatile analysis using HS-SPME–GC–MS

For each beer analyzed, 3.5 mL of sample were deposited in a vial with 1.4 g of NaCl. These vials have a magnetic lid for the SMPE-GCMS technique. The analyses were performed using the Thermo TSQ DUO equipment, under the following conditions: HEADSPACE-SPME: Samples were incubated for 20 min at 30°C with agitation, using DVB/CAR/PDMS fibers. Fiber cleaning was performed at 250°C for 2 min before incubation and after injection, for each sample. GC/MS conditions: The injector was set to 200°C and operated in split-less mode with helium as the carrier gas at a flow rate of 1 mL/min. Column temperatures program (RTX-5 ms, Restek) was: 7 min at 35°C, then ramped to 200°C at 8°C/min and hold 5 min, then ramped to 250°C at 20°C/min. The mass range evaluated was 20–350 m/z with a transfer line temperature of 280°C and ionization source temperature of 250°C.

Standards used: 1-Butanol, 3-methyl-acetate, Ethyl butyrate, Ethyl 2-methyl butyrate, Ethyl acetate, 2,4,6 Trichloroanisole, Ethyl 3-hydroxyhexanoate, *γ*-Decalactone, 2,3 Butadione, Butyric acid, 2-Methoxy-4-vinylphenol, 2-butanol and Phenol, 2,4-dichloro were purchased from Merck-Sigma Aldrich (United States).

NIST Mass Spectral Library used: The library’s search for compounds by the library was performed by comparing the mass spectra obtained using the “National Institute of Standards and Technology” software version 2.2.

### 2.13 Genome sequencing

Cells of L261 and L261 col5 max strains were grown from glycerol stocks on YM plates at 28°C. A single colony was cultivated in 50 mL YM at 28°C without agitation for 24 h; 100 mL Erlenmeyer were used. The cells were collected, washed in TE, and its DNA was extracted using a Wizard kit (Promega, United States). DNA samples were submitted to whole-genome sequencing at Beijing Genomics Institute (BGI) using the Illumina HiSeq2500 technology to a sequencing depth of about 100X.

Raw reads were cleaned using the following steps: (1) Remove reads with at least 40% of low-quality (Q ≤ 20) bases, (2) Remove reads with at least 40% of Ns, (3) Remove adapter contamination, and (4). Reads were mapped against the *S. cerevisiae* strain DBVPG6765 genome (Yue et al., 2017) using BWA, after which duplicates were marked using Picard Tools. Variant calling was performed using freebayes v1.3.6 and were further filtered using vcffilter (“QUAL > 1 & QUAL / AO > 10 & SAF > 0 & SAR > 0 & RPR > 1 & RPL > 1”). The effect on the encoded protein sequence of each variant was predicted using SnpEff v4.3. Variants of interest were inspected manually by examining the alignments on a genome browser. Aneuploidies were inspected by examining genome wide sequencing coverage using tools following the pipeline by O’Donnell et al. (2023). Copy number variants were calculated using CNVkit (Talevich et al., 2016).

### 2.14 Ploidy determination by flow cytometry

Ploidy were evaluated using flow cytometer BD FACS Melody (BD Biosciences, Canada) and Fx Cycle stain (Thermofisher, United States) (Supplementary Figure S2; Supplementary material A1). Yeast cells were inoculated into 35 mL liquid YPD medium and incubated overnight at 25°C with 150 rpm of agitation to obtain 5–8×10^6^ cell/ml. Then, 10 mL of culture was centrifuged at 4000 rpm for 3 min and at 10°C, washed twice with distilled water, and 15 mL of 70% (v/v) ethanol was added. It was incubated at room temperature for 30 min and then centrifuged. The cells were washed 3 times with distilled water. The cells were counted under a microscope and 1×10^6^ cells/ml was taken in a microfuge tube and then the fluorophore PI/ARNasa FxCycle™ (Thermofisher, United States). It was incubated in the dark at 25°C for 1 h. Finally, cells were filtered with a 40 uM cell strainer Falcon™. *S. cerevisiae* BY4741 (haploid) and BY4743 (diploid) were used as control.

## 3 Results

### 3.1 Screening for *Saccharomyces cerevisiae* winemaking strains showing beermaking potential

To select a group of *S. cerevisiae* strains exhibiting promising brewing traits to subjected to an adaptive evolution process, 50 yeast strains were screened for their ability to consume maltose and maltotriose in malt extract wort. These yeasts were isolated from vineyards from different geographical places in Chile, and are part of Kayta’s company culture collection in collaboration with LAMAP-University of Santiago of Chile (Supplementary Table S3; Supplementary material A1). All strains evaluated were able to consume maltose, but only 22 of them consumed maltotriose (Supplementary Table S3; Supplementary material A1). We selected 13 strains due to their higher maltotriose (7 g/L) and maltose (16 g/L) consumption (Supplementary Table S3, marked strains; Supplemental material A1). Furthermore, the results indicated that strains L-3536, L-515, and L-166 consumed 20% of maltotriose, which is 50% less than that of the selected yeasts. However, their maltose consumption was only 10, 80% less than the selected yeasts. This finding is notable because maltotriose, a trisaccharide composed of three glucose units, is generally considered more difficult for yeasts to metabolize than maltose, a disaccharide consisting of two glucose units.

### 3.2 Fermentation traits analysis of the selected strains in synthetic wort supplemented with high maltose concentration

A further selection of yeast strains was performed on synthetic wort to evaluate an array of traits relevant for brewing. The fermentative fitness of 13 strains was evaluated using synthetic wort (density of 1,060 g/mL). In this phase, the 12jj strain was promptly discarded due to its slow growth. We observed higher maltose consumption (around 53 g/L) in cultures inoculated with L718, L168, L159, L167, L249, or L170 strains. Additionally, higher maltotriose consumption (around 2 g/L) was observed in cultures where L440, L512, L2890, L718, L169, or L170 strains were used. Moreover, we observed the highest attenuation by the L261 strain (72.5%), and this strain had one of the highest ethanol productions (Table 1). Glucose and fructose present in wort were depleted by all strains evaluated. In general, all strains produced around 2 g/L of glycerol, and around 0.2 g/L of acetic acid (% CV *<* 10), although the L2890 strain showed higher acetic acid production, reaching 0.3 g/L. Additionally, sensory evaluation tests were carried out on the cultures evaluated. We performed these tests by judges certified by the Beer Judge Certification Program (BJCP). The judges added to the evaluation test two parameters: aromas and tastes (Supplementary Figure S3; Supplementary material A1). The judges evaluated beers produced by L169, L170, L718, 8jj, L261 and L167 strains, highlighting their differentiating aromas and tastes. The judges agreed that these descriptors could generate beers different from currently available commercial beers. Finally, we selected five strains to subject them to an adaptive evolution process (L-170, L-261, L-718, L-169, and L-167); these strains produced beer showing the highest attenuation (around 64%), and ethanol production (around 3% v/v). Moreover, these strains showed the highest maltotriose (2 g/L) and maltose (53 g/L) consumption. Additionally, we selected the 8jj strain for its innovative flavor, as it was highlighted by the judges during the sensory analysis. Therefore, the strain 8jj strain was also subjected to adaptive laboratory evolution.

**Table 1:** Fermentative behaviour of the 12 strains evaluated in synthetic malt wort.

Strains	Maltotriose consumption g/l	Maltose consumption g/l	Glycerol production g/l	Ethanol production %v/v	Attenuation (%)
Synthetic Wort	32.0 ± 0.01	55.3 ± 0.00	0.25 ± 0.01	0 ± 0.00	–
L-440	2.4 ± 0.5 ^a^	44.4 ± 6.9 ^a^	1.69 ± 0.1 ^a^	2.2 ± 0.2 ^a^	51.0 ± 5.7 ^a^
L-512	2.4 ± 0.2 ^a^	50.2 ± 0.5 ^a^	1.9 ± 0.0 ^b^	3.1 ± 0.1 ^b^	61.2 ± 5.7 ^a^
L-2890	2.0 ± 0.2 ^a^	52.7 ± 0.1 ^b^	1.7 ± 0.0 ^a^	2.6 ± 0.4 ^a^	54.1 ± 4.3 ^a^
L-261	1.2 ± 0.1 ^b^	49.4 ± 2.3 ^a^	2.1 ± 0.0 ^c^	3.8 ± 0.2 ^c^	72.5 ± 1.4 ^b^
L-718	1.9 ± 0.1 ^a^	53.5 ± 0.1 ^c^	1.8 ± 0.0 ^d^	2.8 ± 0.0 ^a^	63.3 ± 0.0 ^a^
L-169	2.8 ± 0.1 ^a^	53.5 ± 0.1 ^c^	1.7 ± 0.0 ^a^	3.5 ± 0.6 ^c^	64.3 ± 1.4 ^a^
L-159	1.2 ± 0.5 ^b^	53.3 ± 0.1 ^c^	1.6 ± 0.0 ^a^	2.4 ± 0.2 ^a^	65.3 ± 2.9 ^a^
L-186	1.1 ± 0.7 ^b^	52.0 ± 0.3 ^b^	1.9 ± 0.0 ^b^	3.7 ± 0.4 ^c^	53.5 ± 1.2 ^a^
L-8JJ	1.5 ± 0.8 ^b^	38.0 ± 2.0 ^a^	1.7 ± 0.0 ^a^	2.6 ± 0.4 ^a^	43.0 ± 1.0 ^c^
L-167	0.5 ± 0.0 ^c^	53.2 ± 0.0 ^c^	1.8 ± 0.1 ^a^	3.4 ± 0.2 ^c^	54.4 ± 0.0 ^a^
L-249	0.3 ± 0.0 ^c^	53.2 ± 0.0 ^c^	1.8 ± 0.0 ^a^	3.7 ± 0.3 ^c^	57.0 ± 1.2 ^a^
L-170	2.3 ± 0.1 ^a^	53.1 ± 0.0 ^c^	1.7 ± 0.0 ^a^	3.1 ± 0.2 ^a^	64.0 ± 3.7 ^a^

Equal letters in the same columns indicate that there are no statistically significant differences between the samples, using Mood’s non-parametric test (*p*-value ≤0.05).

### 3.3 Determination of fermentation kinetics of each evolved yeast using synthetic wort supplemented with high maltose concentration

An adaptive evolution process on synthetic wort was performed to improve brewing traits of the selected five strains. To create a strain capable of consuming maltose as a carbon source, maltose concentration was increased at three points during the evolution process. At the end of each of the three phases of the evolution process (described in Materials and Methods), we isolated 3 to 10 colonies per plate. In each test round, the strains showing the shortest lag phase were selected to continue the adaptive evolution process [growth kinetics shown in (Supplementary Figure S4; Supplementary material A1)]. Finally, strains that showed significantly higher growth than the parental strain were selected. Therefore, evolved colonies originating from the parental strains L261 (designated L261 col5 max), L169 (designated L169 max), and 8jj (8jj designated max) were selected. The name scheme was based on the highest growth of the group under evaluation (max). In the case of L261, where several colonies showed similar high growth, we randomly selected colony number 5 (col5). These strains are called “evolved strains” hereafter.

### 3.4 Evolved strains fermentation traits in natural wort

We evaluated the three evolved strains in natural Pale Ale wort. In addition, we used the commercial brewing strain “Nottingham” as a fermentation control. We carried out fermentation experiments and determined the following parameters: sugar consumption, ethanol production and attenuation. Pale Ale wort initially contained 65.6 g/L of maltose, 25.1 g/L of maltotriose, 12 g/L of glucose, and 3.8 g/L of fructose. All the strains depleted the glucose and fructose present in the wort. Interestingly, the L261 col5 max strain consumed higher levels of maltose and maltotriose than the other evolved strains. Likewise, higher ethanol production and attenuation (74%) were observed for the L261 col5 max strain (Table 2).

**Table 2:** Consumption of non-reducing sugars and ethanol production of the evolved strains obtained and the commercial beer brewing strain Lallemand Nottingham™.

Strains	Maltotriose consumption g/l	Maltose consumption g/l	Ethanol production ethanol % v/v	Attenuation % (*)
Natural wort	25.1 ± 0.0	65.6 ± 0.0	0.0 ± 0.0	–
8JJ max	0.0 ± 0.0 ^b^	26.5 ± 0.5 ^a^	1.6 ± 0.3 ^b^	62^b^
L169 max	0.2 ± 0.3 ^a^	36.8 ± 1.1 ^b^	3.1 ± 0.1 ^c^	68^d^
L261 col5 max	7.9 ± 1.1 ^d^	45.1 ± 0.4 ^c^	4.0 ± 0.2 ^d^	74^e^
Nottingham	3.6 ± 1.6 ^c^	46.2 ± 0.5 ^c^	3.2 ± 0.5 ^c^	71^f^

(*) %CV<1%. Equal letters in the same columns indicate that there are no statistically significant differences between the samples, using Mood’s non-parametric test (*p*-value ≤0.05).

Additionally, we determined the flocculation percentage by applying the Helm’s test (Bendiak, 1994), obtaining 78% for L261 col5 max and control strains (commercial strain Nottingham™). Then, the L261 col5 max strain was selected for further evaluation.

### 3.5 Sensory analysis of beers produced by evolved strain

**Figure 2:**
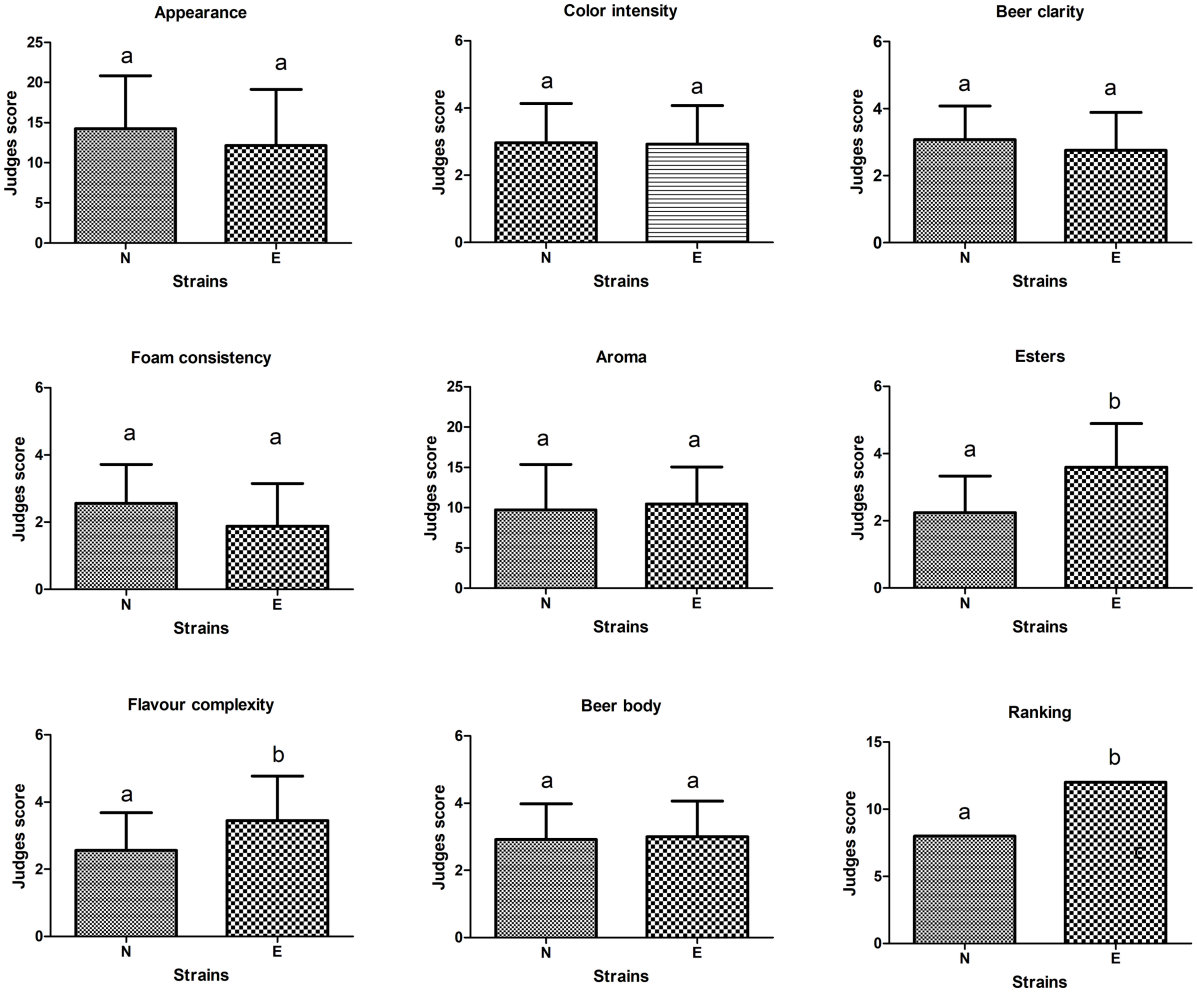
Scores obtained in sensory analysis carried out on beers produced by L261col5 max “evolved” strain (E) compared to Lallemand Nottingham™ (N) control strain. Letters “a” and “b” indicate the existence of differences when statistically comparing beers tasted using Mood’s non-parametric test; therefore, equal letters is indicative that there are no statistically significant differences (*p*-value <0.05).

A hedonic test was performed using a 1-to-5 scale. The details of this scale are indicated in Supplementary Table S2 and Supplementary material A1. This analysis compared the beers produced by the evolved strain L261 col5 max and the control. The parental strain L261 was not evaluated because it was not able to grow under brewing conditions. According to the judge’s opinion, beers produced by L261 col5 max strain showed higher scores in esters complexity compared to the control strain, the commercial beer brewing strain Lallemand Nottingham™ (Figure 2); these differences were statistically significant (Mood test, *p* < 0.05). Interestingly, the other sensory attributes evaluated did not show statistically significant differences. In addition, the preference test placed at 1st place beers produced by L261 col5 max strain (Figure 2).

### 3.6 Beer volatile analysis using HS-SPME–GC–MS

Volatiles produced in beers done by the evolved and control commercial strains were analyzed by GC–MS, using 12 standard compounds and compounds present in the library. Only four of the twelve standard compounds were detected, which were also quantified. The beers produced by the L261 col5 max strain had a greater presence of “green” odors such as green apple (Ethyl 2-methyl butyrate), green grass (Ethyl acetate), cidre (2-Methylbutyl octanoate, 2-methylbutyl ester), green fruity (Ethyl trans-4-decenoate) and citrus (Linalool (terpene)) compared to beers produced using the Nottingham™ strain. Additionally, the evolved strain produced more fatty alcohol than the commercial Nottingham strain, and showed particular odors such as sarsaparilla (2-Undecanol (fatty alcohol)) and floral oily (1-Hexadecanol, 2-methyl-(fatty alcohol)), which demonstrates its aromatic differentiation (Table 3). In contrast, the beers produced by the Nottingham strain mainly showed the presence of compounds related to apricot pear (Octanoic acid, ethyl ester), floral rose, (Acetic acid, 2-phenylethyl ester), and green banana (Hexanoic acid, ethyl ester) (Table 3).

**Table 3:** Compound identified and quantified in beer produced with natural wort using GC–MS and NIST library.

Compound identified and quantified using GC–MS						
RT	Threshold (*)	CAS Number	Odor (*)	Compound	Commercial beer brewing strain Lallemand Nottingham™	The evolved strain L-261 col5 max
	mg/l				mg/l	mg/l
11.02	0.2	123-92-2	Sweet fruity banana	1-Butanol, 3-methyl-, acetate	1.54 ± 0.71^a^	2.37 ± 0.62^a^
8.39	0.001	105-54-4	Fruit pineapple	Ethyl butyrate	5.15 ± 0.62^a^	0.58 ± 0.2^c^
10.07	0.01	7452-79-1	Green apple fruity	Ethyl 2-methyl butyrate	0.32 ± 0.18^a^	0.76 ± 0.18^b^
3.44	5.0	141-78-6	Ethereal fruity sweet weedy green	Ethyl acetate	1.41 ± 0.28^a^	2.84 ± 0.43^b^

Compound identified using NIST library						
RT	Score	CAS Number	Odor	Compound	Commercial beer brewing strain Lallemand Nottingham™	The evolved strain L-265 col5max
10.88	935	624-41-9	Sweet banana, fruity, fruit note	1-Butanol, 3-methyl-, acetate	X	X
14.30	879	123-66-0	Sweet fruity, pineapple, green banana	Hexanoic acid, ethyl ester	X	
16.58	796	78-70-6	Citrus, woody blueberry	Linalool (terpene)		X
18.53	908	106-32-1	Fruity, sweet, apricot banana, pear	Octanoic acid, ethyl ester	X	
19.47	931	103-45-7	Floral rose, sweet honey, fruity tropical	Acetic acid, 2-phenylethyl ester	X	
20.40	814	1653-30-1	Sarsaparilla	2-Undecanol (fatty alcohol)		X
20.31	892	123-29-5	Fruity, rose, wine, tropical	Nonanoic acid, ethyl ester	X	
21.69	871	76649-16-6	Green fruity, cognac, pear note	Ethyl trans-4-decenoate		X
21.83	896	67233-91-4	Fruity fatty	Ethyl 9-decenoate	X	X
21.98	919	110-38-3	Sweet, fruity, apple, grape, brandy	Decanoic acid, ethyl ester	X	X
22.82	878	2035-99-6	Sweet, fruity, pineapple coconut	Octanoic acid, 3-methylbutyl ester		X
22.87	837	67121-39-5	Green apple, cidre	2-Methylbutyl octanoate,2-methylbutyl ester		X
25.49	671	2306-91-4	Banana fruity sweet cognac green	Pentadecanoic acid, 3-methylbutyl ester		X
26.03	716	2490-48-4	Floral oily	1-Hexadecanol, 2-methyl-(fatty alcohol)		X

(*) Odor and threshold descriptions were obtained from Web Book of the National Institute of Standards and Technology-NIST (https://webbook.nist.gov/), the Good Scents Company database (http://www.thegoodscentscompany.com/search3.php), the NCBI database (https://pubchem.ncbi.nlm.nih.gov/), and the Leffingwell & Associates Flavor Database (http://www.leffingwell.com/flavbase.htm).

### 3.7 Genomics analysis

To explore the genetic mechanisms underlying the differences observed in beers produced by the parental L261 and the evolved L261 col5 max strains, we performed whole genome sequencing of both strains using Illumina technology. Initial inspection of novel variants appearing in the evolved strain showed a high number of sites that changed from a heterozygous state in the parental strain (9,163 sites) to either homozygous reference (4,053 sites) or homozygous alternative (4,305 sites) in the evolved strain. This strongly suggested the complete loss of a genome copy (haplotype) during the adaptive evolution process. We further examined alleles with loss of function mutations in a heterozygous state in the parental strain to determine which allele the evolved strain inherited (Supplementary Table S1; Supplementary material A2). Thirteen genes were found to be heterozygous for a non-functional allele in the parental strain but homozygous for the functional allele in the evolved strain. These included genes involved in nutrient transport, utilization and biosynthesis in starving condition such as *HXT13, AGP3* and *VBA2* genes, respectively (Kocaefe-Özşen et al., 2022). Moreover, we identified genes related to lipid biosynthesis (*SPT23*), and genes encoding Chromatin Remodeling Factors related to DNA repair such as *RSF1* (Lans et al., 2012; Min et al., 2013; Price and D’Andrea, 2013); and genes associated with adaptation to stressful or changing environmental conditions (*SSQ1, DOG1, PKR1*).

Interestingly, genes involved in Pheromone-Regulated Membrane protein (*PRM7* and *PRM9*), related to Gcn4 under starving condition (Ayers et al., 2020), were inherited as functional allele in the evolved strain.

Instead, the evolved strain inherited 11 alleles that had loss of function mutations. These included the glycogen debranching enzyme codified by *GDB1* (Walkey et al., 2011), a protein with homology Bul1p, involved in down-regulation of the general amino acid transporter Gap1p codified by *BSC5* (Novoselova et al., 2012); and the gene encoding the zinc-finger protein involved in transcriptional regulation of genes required for glycerol consumption codified by *RSF2* (Böhm et al., 1997; Lu et al., 2005; Tkach et al., 2012). Moreover, *OAF3, SHH4, CFR1* and *HXT11* genes were inherited loss of function alleles. Specifically, *OAF3* gene codes for a putative transcriptional repressor with Zn(2)-Cys(6) finger; which negatively regulates transcription of metabolic pathways involved in fatty acid degradation (MacPherson et al., 2006; Smith et al., 2007; Tkach et al., 2012); *SHH4* gene codes for a putative alternate subunit of succinate dehydrogenase (SDH) (Chang et al., 2015); *CRF1* gene codes for a transcriptional repressor of ribosomal protein (RP) gene transcription, via the TOR signaling pathway-codified (Kumar et al., 2021; Martin et al., 2004), and *HXT11* gene codes a hexose transporter to a broad range of substrates (Ozcan and Johnston, 1999).

Next, we explored novel mutations in the evolved strain. We found only four novel mutations; interestingly, three were located in the *YNL034W* gene, which encodes a protein required for sporulation. However, only one of these three mutations had a predicted change in the protein sequence (Supplementary Table S1; Supplementary material A2).

We inspected whether there were aneuploidies in the evolved strain by checking relative coverage differences across chromosomes. The parental strain did not show any aneuploidy. However, we found 19 aneuploidy events in the evolved strain, with at least one per chromosome, varying from +4 copies at the ends of chromosomes 1 and 6, to +1 copies at the start and ends of chromosomes 4, 11,12 and 13 (Supplementary Table S1; Supplementary material A2). These results were corroborated by analyzing copy number variations between the evolved and parental strains, where we found 226 genes showing higher copy numbers in L261 col5 max, including mostly duplicated copies (Supplementary Table S1; Supplementary material A2). Among these genes, we found duplications in *IMA1*, *MAL13*, and *MAL11*, related with maltose metabolism. We also found 160 genes that lost a copy in the evolved strain, three of which were found at a complete loss: *FLO5*, *PAU8*, and *SEO1*. Moreover, we observed non-synonymous changes in sequence of the *FLO1* gene.

## 4 Discussion

We have applied adaptive evolution to obtain genetic modifications on winemaking yeast strains to achieve and stabilize desired beermaking phenotypes. Evolution results from the gradual accumulation of minor and significant changes in nucleotide sequences in genomes, generating novel alleles or modifying gene activity (Guillamón and Barrio, 2017). This study used Chilean wine yeasts locally adapted to vineyards regions, expecting a significant genetic variation contributing to different outcomes in the adaptive evolution program. Under stress conditions, regional yeast genomes are exposed to dynamic mechanisms that generate genetic polymorphisms with different evolutionary consequences. After 600 generations, we obtained an evolved yeast strain that ferments high gravity wort (29°P), and consuming maltose and maltotriose more efficiently than its parental strain. Through genome sequencing, we observed that the evolved strain suffered the loss of a parental genome and subsequent diploidization, which was confirmed by flow cytometry. Several authors have observed that in fungi the interaction with a stressful environment can lead ploidy changes, which provides an adaptive advantage and permits its evolution (Otto and Whitton, 2000; Zorgo et al., 2013; Dufresne et al., 2014; Todd et al., 2017).

Differences in carbon sources consumption could be related to genes that showed duplications, such *as IMA1, MAL13,* and *MAL11*. The *MAL11* gene encodes a high-affinity transporter of maltose, and it can carry maltotriose, which is consistent with the phenotype obtained. Likewise, the *IMA1* and *MAL13* genes are related to maltose and maltotriose metabolism.

In addition, thirteen genes related to alternative nutrition sources under starvation conditions were homozygous for the functional allele in the evolved strain. These genes are involved in crucial aspects of metabolism, such as nutrient transport and utilization (*HXT13, VBA2,* and *AGP3*), lipid biosynthesis (*SPT23*), regulation of respiration (*RSF1*, and *SSQ1*), and adaptation to changing environmental conditions (*DOG1,* and *PKR1*). Some genes, such as *PRM9* and *PRM7*, have been related to responses to mating signals, which may indirectly affect metabolism in certain contexts. Both genes have consensus sequences for Gcn4p, which is called the general amino acid transcriptional activator, due to under amino acid starvation this protein predominately regulated amino acid biosynthesis (Meimoun et al., 2000; Ayers et al., 2020). Having two functional alleles of these genes may have allowed the evolved strain to optimize its metabolism and compensated for the lack of nutrients.

Interestingly, the genes *RSF1* and *RSF2* have been described as complementary in the pathways for metabolizing non-fermentable sugars such as glycerol as a carbon source under low glucose concentration conditions. These genes encode for proteins that differ in their structure (Lu et al., 2003; Roberts and Hudson, 2009; Yu et al., 2023). Likewise, Rsf1p can regulate these genes, and it can regulate Rsf2 (Yu et al., 2023). The evolved strain was homozygote and inherited only the functional allele of the *RSF1* gene, which could be sufficient to optimize non-fermentable sugar consumption.

In contrast, the evolved strain inherited the non-functional allele of the *CFR1* gene, becoming homozygous strain for this gene. The *CFR1* gene is essential for the survival of the wine yeast *S. cerevisiae* under stress conditions since it can repress the transcription of genes related to the TOR and PKA pathways. These pathways, in turn, allow nutrient consumption, protein biosynthesis, and growth, consuming assimilated carbon and nitrogen sources, such as glucose and glutamine (Zurita-Martinez and Cardenas, 2005).

Besides, the evolved strain inherited the non-functional allele of the *IES5* gene, which is part of the INO80 complex (INO80C), an important regulator of metabolism in eukaryotes in general and in yeasts of the *S. cerevisiae* species in particular (Yuan et al., 2024; Oberbeckmann et al., 2021). Several authors have demonstrated that (INO80C) contributes to coordinating nitrogen and carbon metabolism under stress, and regulates chromatin remodeling for stress adaptation (Tosi et al., 2013; Gowans et al., 2018; Yuan et al., 2024). Under osmotic stress, PKA genes are upregulated, increasing glucose uptake and coordinating metabolic homeostasis (Baccarini et al., 2015; Gowans et al., 2018), and under starvation conditions these genes could be repressed by INO80C (Gowans et al., 2018). Likewise, the lack of IES5 protein could affect INO80 complex function by altering its nutrient sensing capacity, yet *S. cerevisiae* fitness is not affected (Gowans et al., 2018). All this information would indicate that the evolved strain would have deregulated the INO80 system, which allows the activation of the transcription of genes related to catabolism and repression of genes related to anabolism in conditions of lack of nutrients.

The evolved strain inherited the non-functional allele of the *OEF3* gene. This gene negatively regulates the transcription of genes related to fatty acid metabolism, especially oleic acid (Smith et al., 2007). Eliminating the *OEF3* gene allows the expression of genes related to fatty acid oxidation, such as *POT1* (Smith et al., 2007). Oleic acid is one of the main fatty acids in yeasts and contributes to stress tolerance in acidic conditions, such as grape must (Guo et al., 2018). In grape must, glucose and fructose are the main carbon sources therefore, wine yeast *S. cerevisiae* preferentially uses these sugars as a carbon source. However, following their depletion, it can utilize other carbon sources including non-fermentable compounds such as oleic acid (Turcotte et al., 2009). We hypothesized that when the non-functional allele of *OAF3* gene is homozygous, it would allow the transcription of genes related to the metabolization of fatty acids as carbon sources.

We observed that three genes were completely lost in the evolved strain*, PAU8, SEO1* and *FLO5*. Genetic variability observed among *S. cerevisiae* yeasts is important for their adaptation to new environments (Peter et al., 2018). The loss of genes related to flocculation, cell wall organization/biogenesis, transport, and cell division has been observed before (Carreto et al., 2011). The authors observed lower expression of *PAU, SEO1, FLO*, and other genes in wine yeast strains and a high expression in yeast strains not related to wine when both were cultured in synthetic must, mimicking an alcoholic fermentation (Carreto et al., 2011). In contrast, studies in beer have observed a duplication of the *PAU8* and *SEO1* genes when the same brewing yeast strains have been used continuously in brewing processes (Large et al., 2020).

*PAU8* and *SEO1* genes are located near each other on the left arm of chromosome I (Nelissen et al., 1997; Luo and van Vuuren, 2009). *PAU* genes are the most prominent gene family in the species *S. cerevisiae*. They all have common DNA motifs including anaerobic induction and aerobic repression binding sites, TATA boxes, and UME6-related motifs (Luo and van Vuuren, 2009). *PAU* genes are highly active in wine yeast strains and have been linked to stress response and cell wall integrity maintenance. Authors have observed that their expression in *S. cerevisiae* is induced by stressful environments such as alcoholic fermentation in wine (Rachidi et al., 2000; Abramova et al., 2001; Marks et al., 2008; Gordon et al., 2009; Luyt et al., 2024) and by cell-to-cell interactions (Tronchoni et al., 2017; Shekhawat et al., 2019). We hypothesize that *PAU* genes are necessary for stress resistance in wine yeasts exposed to high ethanol concentrations. Therefore, losing these genes under beer production conditions, which contain much less ethanol, would not be relevant for survival.

In yeast, *SEO* genes belong to the allantoate transporter subfamily. They are related to the biosynthesis of sulfur-containing amino acids such as methionine (Isnard et al., 1996; Nelissen et al., 1997; Lombardi et al., 2024), which wine yeast strains use to stabilize reactive oxygen species (ROS) levels by redox balancing (Rząd et al., 2024). Oxidative stress is probably not as relevant for the survival of *S. cerevisiae* in wort compared to grape must.

In addition, the evolved strain showed higher flocculation than other strains. This could be related to non-synonymous changes in sequence of the *FLO1* gene. Studies have observed that *S. cerevisiae* adapts to environmental stress by changing its cell wall composition; for example, under nutrient starvation, *S. cerevisiae* cells interact with other cells, resulting in cell aggregation (Verstrepen and Klis, 2006; Zara et al., 2009). This aggregation is mediated by adhesins, the proteins of FLO gene family that allow plasticity of the cell wall. Studies on the *FLO1* gene have observed that it is highly variable between strains, both in expression and sequence, suggesting that flocculation in *S. cerevisiae* is a dynamic and rapidly evolving trait (Smukalla et al., 2008). Some authors have observed that the *FLO1* and *FLO5* genes have a pleiotropic effect on fermentation kinetics, autolysis, viability, and ester production (Perpetuini et al., 2021; Luyt et al., 2024). Interestingly, when the *FLO5* gene was deleted, floral and fruity attributes were perceived as more intense in sparkling wines evaluated (Perpetuini et al., 2021).

Besides, the evolved strain showed higher ester complexity and exotic odors, which makes it different from the commercial strains used. We were able to detect the high presence of Ethyl 2-methyl butyrate, which is at least twice more than beers produced by other strains. This component has the aroma of green apples as a descriptor, which would explain the perception of the judges. Moreover, it was also possible to identify Linalool (terpene), 2-Undecanol (fatty alcohol), Octanoic acid, 3-methylbutyl ester, and 2-Methylbutyl octanoate, which contribute to green and citrus odors. The evolved strain showed higher production of fatty alcohols than the commercial strain. Fatty alcohol biosynthesis is carried out in two enzymatic steps from fatty acyl–CoAs, which are intermediate compounds in storage lipid biosynthesis. The conversion of fatty acyl–CoA into the corresponding fatty alcohols requires two NADPH molecules, producing two NADP+ molecules. These NADP+ molecules could be used in energy obtaining, such as those involved in pyruvate production, potentially increasing yeast survival (Dahlin et al., 2019).

## 5 Conclusion

In this study, we performed an adaptive evolution program using Chilean native wine yeasts as a strategy to obtain brewery yeasts. The evolved obtained strain produced a beer with higher attenuation and ethanol concentration than the parental strain. Likewise, the evolved strain showed higher ester complexity and exotic odors, making it different from the commercial strain used. This work indicates that obtaining strains with specific characteristics is possible using adaptive evolution, a method that generates indirect mutations (non-GMO). We verified that the evolved strain was able to renew its genome by duplicating genes related to nutrient deficiencies and eliminating genes associated with the survival of wine yeasts under the stress conditions of grape must (lower pH, higher glucose and ethanol concentration than must). In addition, we verified that minor genetic modifications such as SNPs and In/Dels could generate phenotypic changes. Finally, this study indicates that by applying adaptive evolution, it is possible to enhance the use of yeasts belonging to non-brewing origins and unrelated technological applications in beer production, which could carry out efficient brewing fermentation.

## Data Availability

The genomic data has been submitted to the NCBI GenBank repository (Genome Submit PRJNA699892): https://www.ncbi.nlm.nih.gov/datasets/genome/?bioproject=PRJNA699892.
