# Supplementary material for: Obtaining new brewing yeasts using regional Chilean wine yeasts through an adaptive evolution program
Source: Front Microbiol. 2025 Jun 16;16:1599904. doi: 10.3389/fmicb.2025.1599904 (PMC12206800; doi:10.3389/fmicb.2025.1599904)
Supplement: Supplementary file 1 [file Data_Sheet_1.pdf]

Supplemental material A1

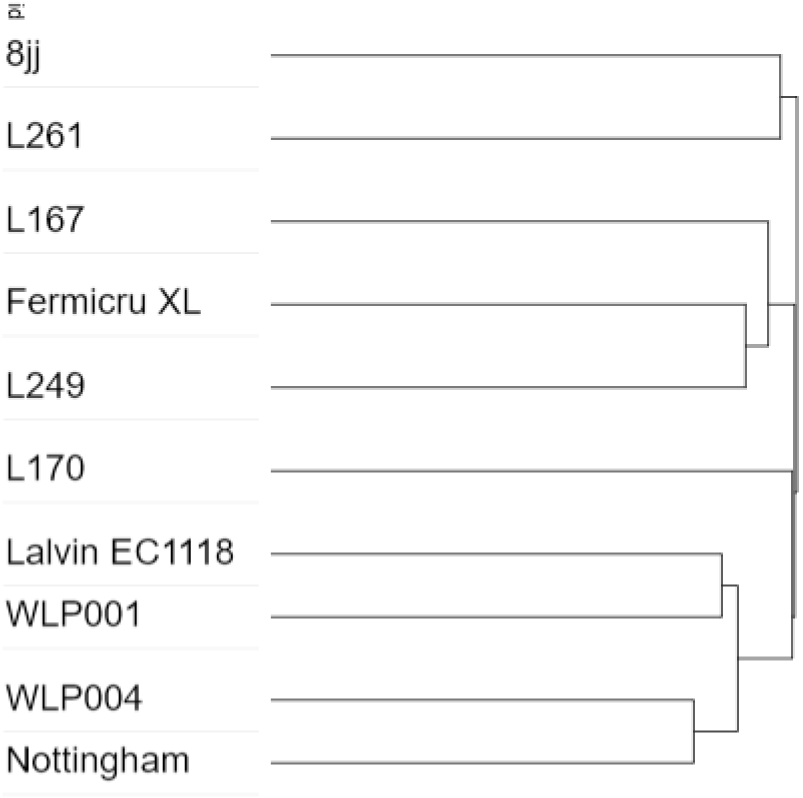

Figure S1. Genetic relationship of Saccharomyces cerevisiae isolates based on the RAPD patterns. The scale bar shows dissimilarity calculated from the Dice coefficient. L261, L170, 8JJ, L169, and L249 were isolated in Chile; Fermicru^{TM} XL (Chilean indigenous character has been confirmed); LalvinTM EC1118 (commercial wine strain); Nottingham^{TM}; WLP001^{TM} and WLP004^{TM} (commercial beer strain).

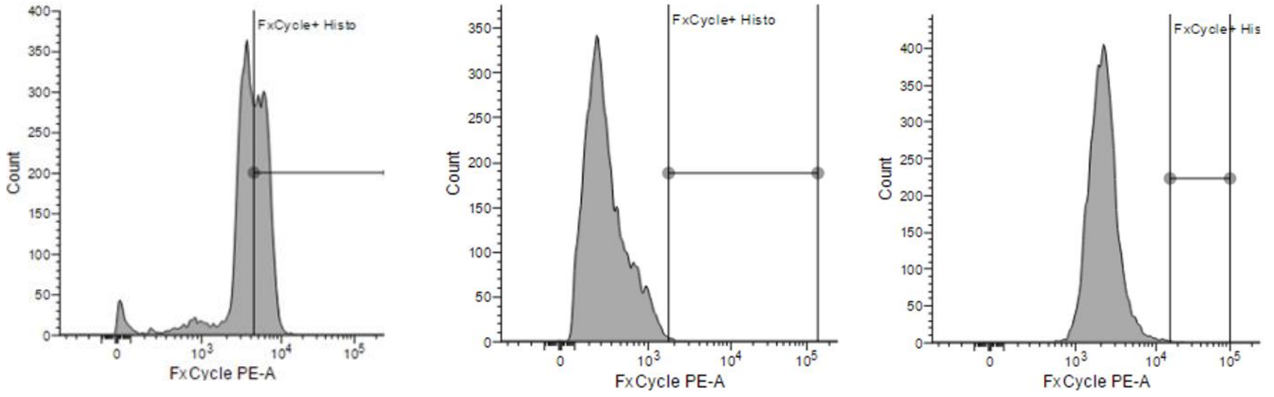

BK37 FxCycle+ BY4741 haploid FxCycle+ BY4743 diploid FxCycle+

Figure S2. Ploidy determination using flow cytometry. Cells were fluorescently labeled using the FxCycle stain.

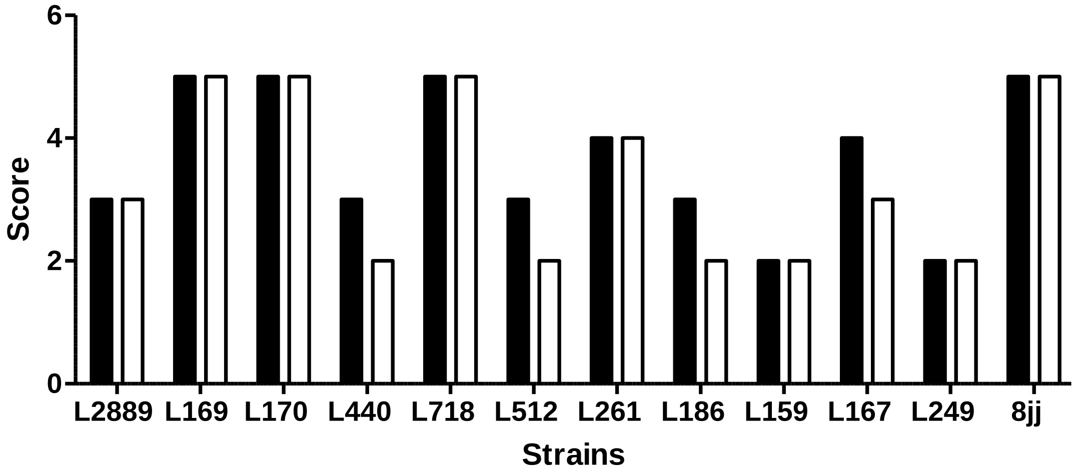

Figure S3. Score of aromas (black) and tastes (white), obtained from the sensory evaluation of fermentations performed by the 12 strains.

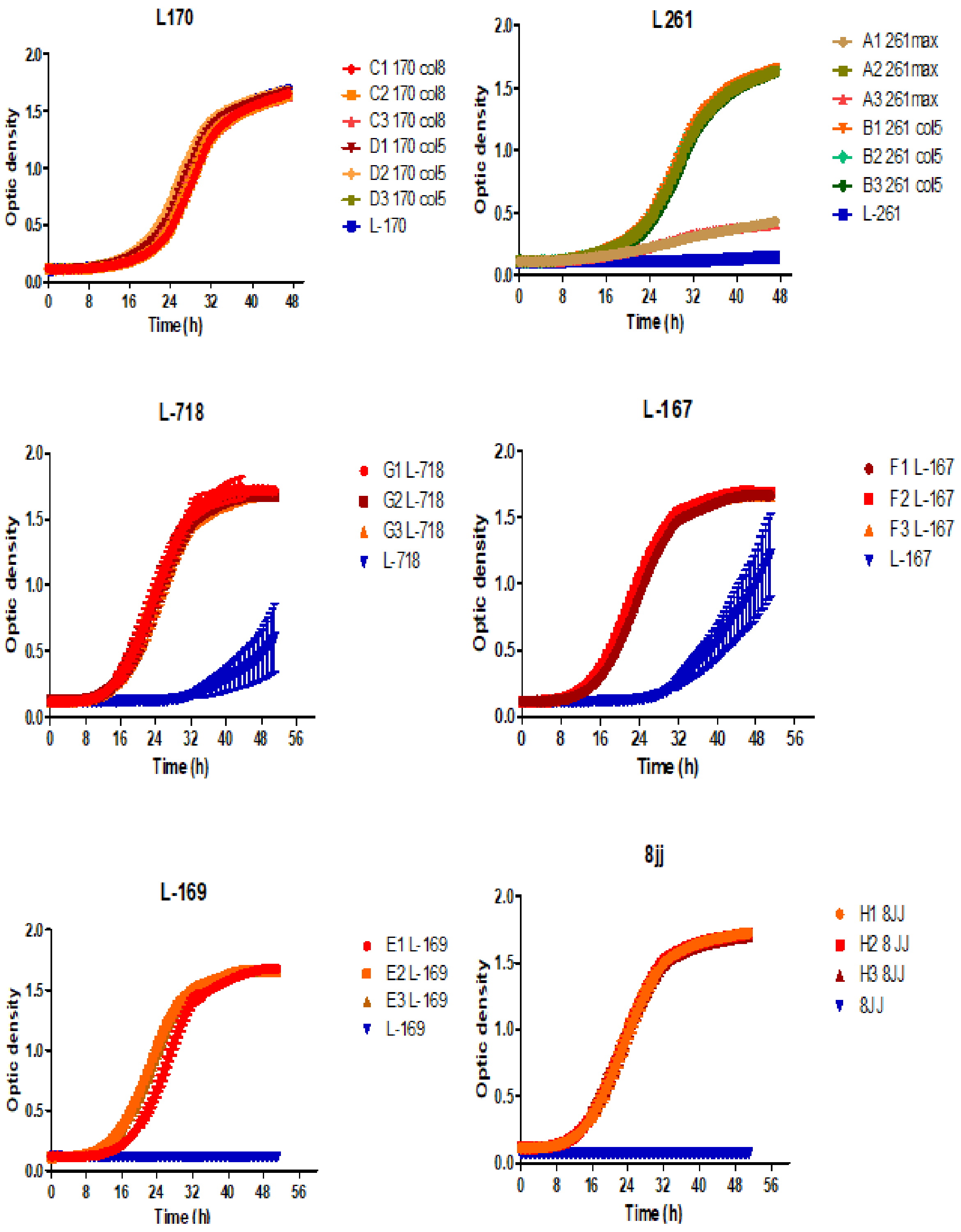

Figure S4. Growth kinetics of evolved colonies 600 generations and the parent strains from which they descend, using synthetic wort supplemented with high maltose concentration.

Table S1. Species and origin of yeasts used in this the study.

N°	Strain	Specie	Region	Place of isolation
**1**	**L-2890**	***Saccharomyces cerevisiae***	**XIII**	**Isla Maipo**
**2**	**L-171**	*Saccharomyces spp*	VIII	Panguilemu
**3**	**L-169**	***Saccharomyces spp***	**VIII**	**Panguilemu**
**4**	**L-508**	*Saccharomyces spp*	VII	Pocilla
**5**	**L-2885**	*Saccharomyces cerevisiae*	VIII	Panguilemu
**6**	**L-170**	***Saccharomyces spp***	**VIII**	**Panguilemu**
**7**	**L-1785**	*Saccharomyces cerevisiae*	VII	Curicó
**8**	**L-529**	*Saccharomyces spp*	VII	Pocilla
**9**	**L-2886**	*Saccharomyces cerevisiae*	VIII	Panguilemu
**10**	**L-2884**	*Saccharomyces cerevisiae*	VII	Santo toribio
**11**	**L-440**	***Saccharomyces spp***	**VII**	**Pichihuerque**
**12**	**L-718**	***Saccharomyces spp***	**XIII**	**Alto Jahuel**
**13**	**L-16**	*Saccharomyces spp*	VII	Cauquenes
**14**	**L-947**	*Saccharomyces spp*	XIII	Alto Jahuel
**15**	**L-512**	***Saccharomyces spp***	**VII**	**Pocilla**
**16**	**L-513**	*Saccharomyces spp*	VII	Pocilla
**17**	**L-288**	*Saccharomyces cerevisiae*	VII	Curicó
**18**	**L-261**	***Saccharomyces spp***	**VII**	**Rincón de Mellado**
**19**	**L-172**	*Saccharomyces spp*	VIII	Panguilemu
**20**	**L-186**	***Saccharomyces cerevisiae***	**VII**	**Pocilla**
**21**	**L-214**	*Saccharomyces spp*	VII	Talca
**22**	**L-244**	*Saccharomyces spp*	VII	Rincón de Mellado
**23**	**L-159**	***Saccharomyces spp***	**VIII**	**Panguilemu**

**24**	**L-160**	*Saccharomyces spp*	VIII	Panguilemu
**25**	**L-162**	*Saccharomyces spp*	VIII	Panguilemu
**26**	**L-163**	*Saccharomyces spp*	VIII	Panguilemu
**27**	**L-167**	***Saccharomyces spp***	**VIII**	**Panguilemu**
**28**	**L-168**	*Saccharomyces spp*	VIII	Panguilemu
**29**	**L-249**	***Saccharomyces spp***	**VII**	**Rincón de Mellado**
**30**	**1jj**	*Saccharomyces cerevisiae*	III	Huasco Alto
**31**	**4jj**	*Saccharomyces cerevisiae*	III	Huasco Alto
**32**	**5jj**	*Saccharomyces cerevisiae*	III	Huasco Alto
**33**	**8jj**	***Saccharomyces cerevisiae***	III	Huasco Alto
**34**	**12jj**	***Saccharomyces cerevisiae***	III	Huasco Alto
**35**	**13jj**	*Saccharomyces cerevisiae*	III	Huasco Alto
**36**	**14jj**	*Saccharomyces cerevisiae*	III	Huasco Alto
**37**	**5DV**	*Saccharomyces cerevisiae*	III	Huasco Alto
**38**	**L-3535**	*Saccharomyces eubayanus*	Patagonia	Argentina
**39**	**L-3534**	*Saccharomyces eubayanus*	Patagonia	Argentina
**40**	**L-3533**	*Saccharomyces eubayanus*	Patagonia	Argentina
**41**	**L-3532**	*Sacvharomyces eubayanus*	Patagonia	Argentina
**42**	**L-3531**	*Saccharomyces eubayanus*	Patagonia	Argentina
**43**	**L-3530**	*Saccharomyces eubayanus*	Patagonia	Argentina
**44**	**L-3529**	*Saccharomyces eubayanus*	Patagonia	Argentina
**45**	**L-3528**	*Saccharomyces eubayanus*	Patagonia	Argentina
**46**	**L-3537**	*Saccharomyces eubayanus*	Patagonia	Argentina
**47**	**L-3536**	*Saccharomyces eubayanus*	Patagonia	Argentina
**48**	**6DV**	*Saccharomyces cerevisiae*	III	Huasco Alto
**49**	**L-515**	*Saccharomyces spp*	VII	Pocilla
**50**	**L-166**	*Saccharomyces spp*	VIII	Panguilemu

Table S2. A hedonic test performed with a score range that varied from 1 to 5. The evaluated attributes were appearance, color intensity, beer clarity, foam consistency, aromas, esters, flavor complexity, and beer body.

Scale	Meanings
1	Attribute intensity is lower
2	Attribute intensity is low
3	Attribute intensity is average
4	Attribute intensity is high
5	Attribute intensity is higher

Table S3. Maltotriose and maltose consumption of fifty *S. cerevisiae* strains grown in YPMM broth.

N°	Strains	Maltotriose consumption (g/l)	Maltose consumption (g/l)	N°	Strains	Maltotriose consumption (g/l)	Maltose consumption (g/l)
**1**	**L-2890***	7,1±0,0	17,0±0,0	**26**	**L-163**	0,7±0,0	16,9±0,1
**2**	**L-171**	0,4±0,0	16,0±0,0	**27**	**L-167***	7,3±0,0	17,8±0,0
**3**	**L-169***	7,1±0,0	17,0±0,0	**28**	**L-168**	0,5±0,0	17,1±0,0
**4**	**L-508**	0,3±0,0	16,1±0,0	**29**	**L-249***	6,1±0,0	17,8±0,0
**5**	**L-2885**	0,2±0,2	16,0±0,2	**30**	**1jj**	4,0±0,4	18,2±0,1
**6**	**L-170***	7,1±0,0	17,0±0,0	**31**	**4jj**	0,1±0,1	17,9±0,0
**7**	**L-1785**	0,2±0,1	16,3±0,1	**32**	**5jj**	0,1±0,1	18,0±0,1
**8**	**L-529**	0,3±0,2	16,0±0,1	**33**	**8**jj*	7,0±0,0	18,5±0,0
**9**	**L-2886**	0,4±0,0	16,3±0,0	**34**	**12jj***	7,0±0,0	18,4±0,0
**10**	**L-2884**	0,2±0,1	17,6±0,0	**35**	**13jj**	3,6±0,1	18,2±0,1
**11**	**L-440***	7,0±0,0	17,7±0,1	**36**	**14jj**	0,2±0,0	17,9±0,0
**12**	**L-718***	7,0±0,0	17,8±0,0	**37**	**5DV**	0,1±0,0	17,9±0,0
**13**	**L-16**	0,8±0,4	17,1±0,1	**38**	**L-3535**	0,5±0,0	17,9±0,0
**14**	**L-947**	5,0±0,3	17,7±0,0	**39**	**L-3534**	0,5±0,1	18,0±0,1
**15**	**L-512***	7,0±0,0	17,8±0,0	**40**	**L-3533**	0,2±0,0	18,2±0,1
**16**	**L-513**	0,2±0,1	16,8±0,0	**41**	**L-3532**	0,2±0,1	18,1±0,3

**17**	**L-288**	1,7±0,4	17,3±0,2	**42**	**L-3531**	0,1±0,0	18,3±0,0
**18**	**L-261***	7,0±0,0	17,7±0,0	**43**	**L-3530**	0,1±0,1	18,3±0,0
**19**	**L-172**	0,2±0,1	17,2±0,2	**44**	**L-3529**	0,2±0,1	18,2±0,0
**20**	**L-186***	7,0±0,0	17,7±0,0	**45**	**L-3528**	0,3±0,1	18,1±0,0
**21**	**L-214**	0,5±0,0	16,8±0,0	**46**	**L-3537**	0,1±0,1	18,2±0,0
**22**	**L-244**	5,8±0,0	17,6±0,1	**47**	**L-3536**	2,0±0,0	2,6±0,2
**23**	**L-159***	7,3±0,0	17,8±0,0	**48**	**6DV**	0,2±0,0	18,0±0,0
**24**	**L-160**	4,2±0,1	17,4±0,0	**49**	**L-515**	2,0±0,1	2,9±0,2
**25**	**L-162**	1,1±0,1	17,3±0,1	**50**	**L-166**	2,1±0,0	3,0±0,1
